# Anticoagulation and antiplatelet therapy in short bowel syndrome: A systematic review

**DOI:** 10.1016/j.intf.2024.100007

**Published:** 2024-07-15

**Authors:** Carolyn Mercer, Anna Crawford, Susan Shapiro, Philip J. Allan, Tim Ambrose

**Affiliations:** a Translational Gastroenterology and Liver Unit, NIHR Oxford Biomedical Research Centre, Oxford University Hospitals NHS Foundation Trust, John Radcliffe Hospital, Oxford OX3 9DU, United Kingdom; b Oxford Haemophilia and Thrombosis Centre, NIHR Oxford Biomedical Research Centre, Oxford University Hospitals NHS Foundation Trust, Nuffield Orthopaedic Hospital, Oxford OX3 7JT, United Kingdom; c Radcliffe Department of Medicine, Oxford University, Oxford OX3 9DU, United Kingdom

**Keywords:** Antiplatelet, Anticoagulant, Anticoagulation, Short bowel syndrome, Short gut, Parenteral nutrition

## Abstract

**Background:**

Short bowel syndrome (SBS) is one cause of intestinal insufficiency or failure. Drug therapy may be difficult in this patient group at least in part due to abnormalities in anatomy or surface area for absorption. Anticoagulation may be indicated due to the underlying cause of SBS or to treat complications of parenteral support.

**Aims:**

To review the literature on the use of anticoagulation and antiplatelet agents in SBS.

**Methods:**

Systematic literature search in MEDLINE, EMBASE and the Cochrane library for studies pertaining to SBS, anticoagulants and antiplatelets to 22nd February 2024.

**Results:**

24 of 725 screened articles met the eligibility criteria. The majority were case reports or series with no randomised controlled trials. Warfarin was the most studied anticoagulant with adequate anticoagulation with gut lengths as short as 12 cm. Rivaroxaban was the most studied DOAC with target plasma concentrations achieved with as little as 30 cm intestine. Apixaban was studied in one case with target plasma concentrations reached and no recurrent thrombosis or bleeding events. Dabigatran was not shown to reliably achieve target plasma concentrations. Minimal data was available on antiplatelet agents in short bowel syndrome with no long term clinical outcomes reported.

**Conclusions:**

The literature is largely limited to case reports and series. Warfarin is the most widely studied anticoagulant in SBS and is effective. Absorption of rivaroxaban and, probably, apixaban is adequate in SBS but longer term studies on clinical outcomes are needed. Insufficient data is available to make firm recommendations for antiplatelet therapy.

## Introduction

### Rationale

Intestinal failure may be defined as “the reduction in gut function below the minimum necessary for the absorption of macronutrients and/or water and electrolytes, such that intravenous supplementation is required to maintain health and/or growth”. [1] Intestinal insufficiency refers to a reduction in gut absorptive function that does not require intravenous supplementation. One cause of intestinal failure/insufficiency is short bowel syndrome (SBS) typically defined as a length of small intestine in continuity of less than 200 cm. [1] Drug therapy in patients with SBS can be challenging due to altered anatomy and surface area available for absorption. However, there are other concomitant factors that may influence drug absorption and metabolism in SBS patients including mucosal integrity (e.g. Crohn’s disease), dysbiosis, intestinal motility (e.g. enteric dysmotility), reduced transit time, site of drug absorption, pH, and adaptive changes in enterocytes following bowel resection. [2] As a result, drug serum levels and clinical effect can be unpredictable.

Patients with SBS may require long term anticoagulation or antiplatelets as a consequence of the underlying aetiology for their short bowel, such as superior mesenteric artery (SMA) thrombosis; or due to associated clinical complications, such as catheter-related venous thrombosis in the setting of permanent central venous access; or because of other indications such as atrial fibrillation, ischaemic heart disease or peripheral vascular disease. Specifically for mesenteric ischaemia, the decision about anticoagulation or antiplatelet therapy may depend on the underlying aetiology. For example, embolic phenomena secondary to atrial fibrillation would likely be managed with anticoagulation whereas intrinsic thrombotic events may be best managed with antiplatelet therapy. Historically warfarin and aspirin have been the oral anticoagulant and antiplatelet of choice in patients with SBS, albeit with limited evidence to support their efficacy. In recent years direct oral anticoagulants (DOACs) have emerged as first line agents for the treatment and prevention of venous thromboembolism (VTE) and stroke prevention in patients with atrial fibrillation [3], and low dose rivaroxaban in conjunction with aspirin is now licensed for secondary prevention in patients with stable atherosclerotic disease [4]. Although subcutaneous low molecular weight heparin (LMWH) can be prescribed for long term anticoagulation the only evidence for its longterm effectiveness is in cancer associated VTE [5], and it can be burdensome for patients and carries the risk of skin hypersensitivity reactions and osteoporosis. [6] If however, an injectable form of anticoagulation is required fondaparinux may be more appropriate because it carries a lower risk of osteoporosis in the long term compared to LMWH. [7], [8].

Dosing of warfarin to maintain a therapeutic target international normalised ratio (INR) in patients with SBS can be challenging due to fluctuating vitamin K levels, bacterial overgrowth and hepatic impairment. [9] In contrast to warfarin, DOACs have more predictable pharmacokinetics, their mechanism of action is not influenced by dietary vitamin K and they are not susceptible to numerous drug interactions. [3], [10] They are given in fixed dosing regimens eliminating the need for intensive monitoring and dose titration that warfarin requires. [10] When given in fixed dose, DOAC levels vary in different people and the 90 % CI intervals for expected peak and trough levels are wide. [11], [12], [13] Although expected peak and trough ranges may give an indication that someone has a reasonable plasma level of DOAC, evidence for clinical efficacy is not linked to particular peak/trough target levels. DOACs are only routinely recommended in people with good bowel absorption. However certain DOACs such as rivaroxaban are absorbed from the proximal gastrointestinal tract [14] and therefore could potentially have reasonable absorption profiles in patients with SBS.

Newer antiplatelet agents such as ticagrelor and prasugrel have been used routinely in clinical practice for many years. The different pharmacokinetic and pharmacodynamic profiles of these medications make them welcome additions to the therapeutic armamentarium for medical practitioners. The properties of these newer antiplatelet agents have also been advantageous in overcoming limitations of traditional aspirin and clopidogrel therapy, such as inter-individual variability resulting from CYP450 polymorphisms. [15].

With these novel anticoagulant and antiplatelet agents being widely adopted in clinical practice it is timely to review the evidence for their use in patients with SBS.

### Objectives

The objective of our study was to systematically review the literature for the quantitative evidence of the efficacy of anticoagulation and antiplatelets in patients with SBS.

## Methods

Reporting of this study adhered to the Preferred Reporting Items for Systematic Reviews and Meta‐Analyses (PRISMA) statement. [16].

### Eligibility criteria

To be included in the review, papers needed to focus on the efficacy of antiplatelets or anticoagulation in patients with SBS. Case reports, case series, conference abstracts, cohort studies, cross-sectional studies and clinical trials were eligible for inclusion. Non-clinical, non-human and studies restricted to paediatric populations were excluded.

### Search strategy

A systematic search of medical literature up to 22nd February 2024 was carried out by an experienced librarian using MEDLINE, EMBASE and the Cochrane library. Studies were identified using the following MeSH terms ‘short bowel syndrome* ’, ‘anticoagulants* ’ and ‘antiplatelets* ’ plus keywords and synonyms derived from them (please see Supplementary Methods for full search strategy). Reference lists of eligible articles, and any review articles, were also reviewed to ensure all relevant literature was captured.

The search strategy was filtered to human studies only. Recognising that the literature base was likely to be small no study design, date or language restrictions were imposed as we did not want to miss any relevant articles. Although non-English articles were not planned to be translated in full, oftentimes they have an English abstract and we hoped to extract relevant data from this even if the whole article could not be assessed.

### Study selection

Three investigators (CM, AC, TA) independently reviewed the results from the searches on electronic databases to identify articles relevant to the review based on the abstract or abstract and article. Studies were assessed according to the predefined eligibility criteria and included only with unanimous agreement between the three investigators. If there was any disagreement between investigators this was resolved with review by a fourth investigator (PA).

### Data extraction and management

Data was extracted independently by two authors (CM & TA) in duplicate, according to a predefined protocol and recorded in a Microsoft Excel spreadsheet. The following variables were extracted from each eligible publication: First author name, year of publication, study design, number of participants, aetiology of short bowel syndrome, gut anatomy of study participants, indication for anticoagulation or antiplatelets agent, agent used, intervention if relevant including type, dose, mode of delivery and timing of sampling, and outcomes.

Additional outcomes of interest were dependent on the medication. For participants taking warfarin the main outcomes of interest were achieving and maintaining a therapeutic INR and time in therapeutic range. For those patients taking a DOAC the main outcomes of interest were recurrent VTE, plasma concentrations (Cmax, steady state concentration) in comparison to normal population data and adverse events. Outcomes of interest in patients taking antiplatelet agents included recurrent thrombotic events, such as in-stent thrombosis following coronary/mesenteric artery stenting or recurrent mesenteric ischaemia, and platelet function testing. Whilst conceivable that some publications might include patients on both antiplatelet and anticoagulant agents no additional or different outcomes were pre-specified.

### Assessment of study quality

Of 24 included studies 19 were non-comparative, descriptive case reports or case series. Formal assessment of these was not performed. Four studies were cohort studies – two measured pharmacokinetic data of DOACs in short bowel syndrome, one measured time in the therapeutic range for warfarin, and one assessed platelet function with aspirin. None directly compared interventions or exposures and so study quality was not performed. The final study was an interventional crossover study to look at pharmacokinetic data rather than objective markers of effectiveness of DOACs and as such was not formally assessed for quality.

No missing data related to the outcomes of interest were identified but where anatomical data was not clear from the paper the authors were contacted, where possible, and additional information included if available.

### Data synthesis and statistical methods

Table 1 summarises the characteristics and extracted data variables of the publications included in the systematic review. A meta-analysis was not performed due to both the heterogeneity of the studies and the lack of randomised control trials.

**Table 1:** Summary of studies included in analysis.

Article	Design	Medication	Aetiology of Intestinal Failure	Anatomy (Length of small bowel in cm)	Colon in continuity	Indication for Anticoagulation/Antiplatelet	Outcome
Mitchell *et al.*, 1977	Case report	Warfarin	Mesenteric ischaemia (arterial)	100 cm	Yes (full)	PE	Achieved therapeutic INR
Lehman *et al.*, 1985	Case report	Warfarin	Perforated diverticular abscess with bowel resection	15 cm	No	DVT	Achieved therapeutic INR
Lutomski *et al.*, 1985	Case series	Warfarin	Multiple bowel resections	Not documented	Yes (length of residual colon unknown)	Line thrombus	Achieved therapeutic INR
		Warfarin	Mesenteric ischaemia	25 cm (ileum)	Yes (full)	PE	Achieved therapeutic INR
		Warfarin	Mesenteric ischaemia	Proximal jejunum	Yes (partial)	Line thrombus	Achieved therapeutic INR
		Warfarin	Mesenteric ischaemia	15 cm	Yes (partial)	Line thrombus	Achieved therapeutic INR
		Warfarin	Multiple bowel resections (IBD)	12 cm	Unknown	Line thrombus	Achieved therapeutic INR
Kearns *et al.*, 1986	Case report	Warfarin	Mesenteric ischaemia (arterial)	30 cm	Yes (partial)	Mesenteric thrombus	Achieved therapeutic INR
Lutomski *et al.*, 1987	Case report	Warfarin	Multiple bowel resections (IBD)	12 cm	Unknown	Line thrombus	Achieved therapeutic INR
Gimmon *et al.*, 1987	Case series	Warfarin	Mesenteric ischaemia (arterial)	Duodenum + 5 cm jejunum	Yes (partial)	Mural thrombus	Achieved therapeutic INR
		Warfarin	Mesenteric ischaemia (venous)	Duodenum + 40 cm SB	Yes (full)	Mesenteric thrombus	Achieved therapeutic INR
Owens *et al.*, 1990	Case report	Warfarin	Mesenteric ischaemia (arterial)	20 cm	No	Mesenteric thrombus	Achieved therapeutic INR
Brophy *et al.*, 1998	Case report	Warfarin	Bowel resection secondary to multiple gunshot wounds	Not documented	Yes (full)	DVT	Warfarin resistant, unable to achieve therapeutic INR
Batke-Hastings *et al.*, 2008	Case series	Warfarin*	Gastrointestinal dysmotility	Intact SB	Yes	Line thrombus	Achieved therapeutic INR
		Warfarin*	Functional SBS (High output ileostomy)	Intact SB	No	PE	Achieved therapeutic INR
Lopez *et al.*, 2010	Case report	Warfarin	Crohn’s disease	Not documented	Unknown	DVT	Recurrent DVT despite therapeutic INR > 75 % of the time.
Khakwani *et al.*, 2019	Case report	Warfarin	Mesenteric ischaemia (venous)	Complete small bowel resection	No	Mesenteric thrombus	Achieved therapeutic INR
Quintal *et al.*, 2019	Case report	Warfarin	Mesenteric ischaemia (arterial)	45 cm	Yes (partial)	Mesenteric thrombus	Achieved therapeutic INR
Fumagalli *et al.*, 2022	Cohort	Warfarin	Mesenteric ischaemia (n = 7), Peritonitis (n = 5)	Not documented	Not documented	VTE (n = 7), AF (n = 5)	Achieved therapeutic INR with median time in therapeutic range 62 % (comparable to controls)
Theodoulou *et al.*, 2023	Case report	Warfarin* *	Ulcerative colitis but reason for small bowel resection not known	Not documented	No	VTE (DVT, PE, portal vein, splanchnic) and single positive APS	Achieved therapeutic INR with time in therapeutic range 60 %
Inaba *et al.*, 2010	Case report	Heparin	Mesenteric ischaemia (arterial)	Proximal jejunum	No	Mesenteric thrombus	Achieved therapeutic APTT
Pollak *et al.*, 2018	Case report	Apixaban	Mesenteric ischaemia	Duodenum + 30 cm	Unknown	Mesenteric thrombus	Comparable plasma concentrations (Cmax) to population data using personalised dosing
Douros *et al.*, 2014	Case report	Dabigatran/ rivaroxaban	Mesenteric ischaemia (arterial)	Duodenum + 100 cm	Yes	AF	Suboptimal plasma concentrations (Cmax & Ctrough) with dabigatran. Comparable plasma concentrations (Cmax) to population data with rivaroxaban.
Cheung *et al.*, 2017	Interventional crossover	Dabigatran/ Rivaroxaban	Radiation enteritis	140 cm	No	No indication	Higher value-to-reference ratios of rivaroxaban PK parameters (Cmax & Ctrough) compared to dabigatran
		Dabigatran/ Rivaroxaban	Mesenteric ischaemia	50 cm	Yes (length of residual colon unknown)	No indication	Higher value-to-reference ratios of rivaroxaban PK parameters (Cmax & Ctrough) compared to dabigatran
		Dabigatran/ Rivaroxaban	Diverticular perforation/ Mesenteric ischaemia (arterial)	15 cm	No	Mesenteric thrombus	Negligible absorption of both drugs
		Dabigatran/ Rivaroxaban	Mesenteric ischaemia (venous)	35 cm	Yes (length of residual colon unknown)	Protein S deficiency	Higher value-to-reference ratios of rivaroxaban PK parameters (Cmax & Ctrough) compared to dabigatran
		Dabigatran/ Rivaroxaban	Bowel resection (Crohn’s disease)	170 cm	No	APS	Higher value-to-reference ratios of rivaroxaban PK parameters (Cmax & Ctrough) compared to dabigatran
		Dabigatran/ Rivaroxaban	Mesenteric ischaemia	170 cm	Yes (length of residual colon unknown)	No indication	Higher value-to-reference ratios of rivaroxaban PK parameters (Cmax & Ctrough) compared to dabigatran
Christensen *et al.*, 2014	Case series	Rivaroxaban	Mesenteric ischaemia (venous)	Duodenum + 30 cm	Yes (SB anastomosed to ascending colon)	Mesenteric thrombus	Comparable plasma concentrations (Cmax) to population data
		Rivaroxaban	Dysmotility with multiple bowel resections	Duodenum + 75 cm	Yes (partial)	Line thrombus	Comparable plasma concentrations (Cmax) to population data
		Rivaroxaban	Multiple bowel resections (Crohn’s disease)	Duodenum + 100 cm	Yes (partial)	DVT	Comparable plasma concentrations (Cmax) to population data
Bavalia *et al.*, 2019	Cohort	Rivaroxaban	Mesenteric ischaemia	45 cm	Unknown	AF	Suboptimal plasma concentrations (Cmax)
		Rivaroxaban	Refractory obstipation	Not documented	Unknown	VTE	Comparable plasma concentrations (Cmax) to population data
		Rivaroxaban	Mesenteric ischaemia	85 cm	Unknown	AF	Comparable plasma concentrations (Cmax) to population data
		Rivaroxaban	Mesenteric ischaemia	70 cm	Unknown	AF	Suboptimal plasma concentrations (Cmax)
		Rivaroxaban	Refractory obstipation	120 cm	Unknown	VTE	Comparable plasma concentrations (Cmax) to population data
Van Haaps *et al.*, 2022^α^	Cohort	Rivaroxaban	Not documented (short bowel syndrome n = 10, other malabsorption or pseudoobstruction n = 7)	Not documented	Not documented	VTE (n = 16), AF (n = 1)	Mean plasma concentrations (Cmax 203 ng/mL, Cmin 37 ng/mL) enabled continuation of rivaroxaban in n = 14 patients.
Faye *et al.*, 2014	Cohort	Aspirin	Mesenteric ischaemia	Duodenum + 35 cm	No	No indication	Acceptable platelet inhibition at Tmax when measured by LTA
		Aspirin	Mesenteric ischaemia	Duodenum + 80 cm	No	No indication	Acceptable platelet inhibition at Tmax when measured by LTA
		Aspirin	Mesenteric ischaemia	Duodenum + 115 cm	No	No indication	Acceptable platelet inhibition at Tmax when measured by LTA
		Aspirin	Mesenteric ischaemia	Duodenum + 150 cm	No	No indication	Acceptable platelet inhibition at Tmax when measured by LTA
		Aspirin	Mesenteric ischaemia	Duodenum + 50 cm	Yes (partial)	No indication	Acceptable platelet inhibition at Tmax when measured by LTA
		Aspirin	Mesenteric ischaemia	Duodenum + 120 cm	Yes (partial)	No indication	Acceptable platelet inhibition at Tmax when measured by LTA
		Aspirin	Mesenteric ischaemia	Duodenum + 30 cm	Yes (full)	No indication	Acceptable platelet inhibition at Tmax when measured by LTA
		Aspirin	Mesenteric ischaemia	Duodenum + 45 cm	Yes (full)	No indication	Acceptable platelet inhibition at Tmax when measured by LTA
		Aspirin	Mesenteric ischaemia	Duodenum + 70 cm	Yes (full)	No indication	Acceptable platelet inhibition at Tmax when measured by LTA
Hadley *et al.*, 2014	Case report	Aspirin/ clopidogrel/ ticagrelor	Mesenteric ischaemia	Not documented	Unknown	Coronary artery stenting	Acceptable platelet inhibition measured by LTA with aspirin & ticagrelor. Partial response to clopidogrel.
Droppa *et al.*, 2015	Case report	Aspirin/ clopidogrel/ prasugrel/ ticagrelor	Mesenteric ischaemia (arterial)	Duodenum + 30 cm	Unknown	Coronary artery stenting	Acceptable platelet inhibition measured by MEA with aspirin & ticagrelor. Insufficient platelet inhibition with clopidogrel & prasugrel.

Abbreviations/Symbols: AF, atrial fibrillation; APS, antiphospholipid syndrome; IBD, inflammatory bowel disease; LTA, light transmission aggregometry; MEA, multiple electrode aggregometry; PK, pharmacokinetic; SMA, superior mesenteric artery; SB, small bowel; * , sublingual administration; * *, liquid warfarin; α, size of cohort n = 17 and possibly some overlap in cases with Bavalia et al. 2019

## Results of search strategy

### Search

**Fig. 1:**
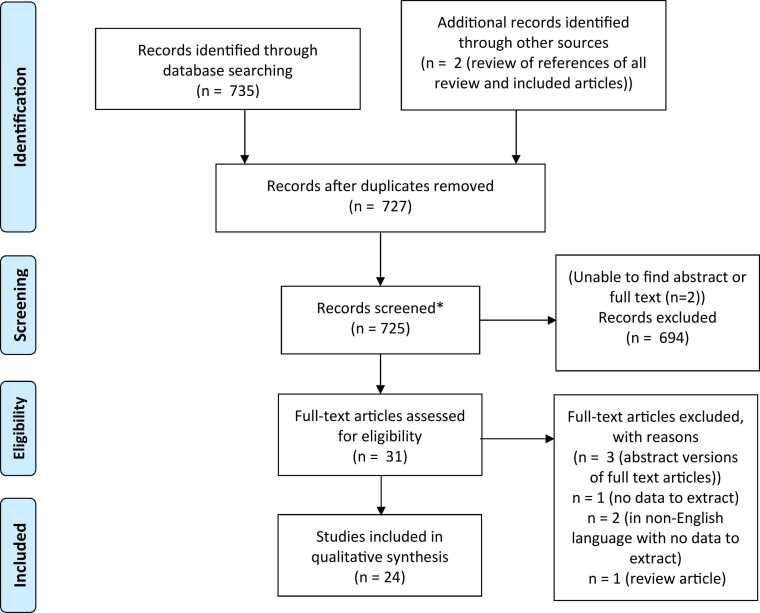
PRISMA flow diagram for assessing eligibility of studies.

After the removal of duplicate records, the search strategy identified 727 unique records including two studies identified after screening reference lists of review articles or included studies. Following screening of the abstract and/or full text, and review of 27 articles by a fourth investigator (PA) due to disagreement about eligibility, a total of 31 articles met the inclusion criteria. Three were subsequently excluded because they were abstract versions of full text articles that had already been screened (i.e. duplicates). Two case reports published in French and Japanese were excluded after more careful review of the English abstract as insufficient data could be extracted to contribute significantly to the review. An additional article was excluded following a more detailed review as there was no primary data to extract, and a final article excluded as it transpired to be a review article. A flow chart summarising the study screening selection is shown in Fig. 1.

Most of the papers identified were case reports or case series describing responses to a particular anticoagulant or antiplatelet agent. There were a small number of cohort studies included in the review. The majority of papers identified examined the use of warfarin in patients with SBS.

## Results and discussion of identified studies

### Warfarin

#### Class of drug and mode of absorption

Warfarin is a water soluble compound which is passively absorbed from the proximal small bowel via a concentration dependent mechanism and is an antagonist of Vitamin K. [17] Historically this absorption profile has made warfarin an excellent choice of oral anticoagulant in patients with SBS. Our literature contains thirty cases of successful anticoagulation with warfarin as evidenced by patients achieving and maintaining a therapeutic target INR. [6], [17], [18], [19], [20], [21], [22], [23], [24], [25], [26].

#### Discussion of warfarin studies

Indication for anticoagulation varied widely amongst the case reports and included pulmonary embolus, deep vein thrombosis, catheter-related venous thrombosis, and mesenteric thrombosis. Time from small bowel resection to initiation of warfarin therapy, where detailed, differed considerably amongst the cases ranging from within one month to several months post-resection. This may potentially be important as it has been postulated that patients undergo intestinal adaptation in the months following surgery, which may result in improved absorption. [19] However the timing of warfarin therapy post bowel resection did not ultimately appear to influence the efficacy of warfarin and the main reason for failure to achieve an adequate INR was attributed to concomitant administration of intravenous vitamin K. [21], [22], [25].

All the case reports used oral warfarin administration (liquid formulation in one case) except for two in which they utilised the sublingual route. [6] Where this was documented, all of the cases were on an intravenous or subcutaneous anticoagulant prior to being commenced on oral warfarin. Three cases were initially started on intravenous coumarin before being transitioned to oral warfarin. [19], [20] Doses required to achieve a therapeutic INR were highly variable, and ranged from 0.5 mg^20^ to 10 mg daily. [19], [22].

All the case reports equated efficacy with achieving a therapeutic target INR. In those cases where the target INR was clearly documented the majority aimed for an INR between 2 and 3. [6], [23], [24], [25] In all of the case reports the patients had their INR monitored on serial blood tests however the follow up period following initiation of therapy varied greatly ranging from 6 days [19] to a median 48 months. [27] Three studies assessed time in therapeutic range (TTR). [23], [27], [28] In one case the patient had SBS in the context of Crohn’s disease (anatomy unknown) and was documented to have a TTR > 75 %. [23] It is however unclear over what time period the TTR was calculated. The current European Guidelines recommend a TTR > 70 % over a period of six months. Despite having a stated TTR of > 70 % the patient developed a DVT. The authors proposed that the patient had a propensity to develop thrombi despite anticoagulation therapy due to their underlying inflammatory bowel disease. [23] The two other studies demonstrated a TTR of 62 %^27^ and 60 %^28^ the former demonstrating no appreciable increase in thrombotic or bleeding episodes compared to matched controls.

There were a small number of case reports that undertook additional pharmacokinetic testing. Lehman et al. collected 24-hour jejunostomy output in a patient with 15 cm of jejunum on two occasions to assay for warfarin content and establish the percentage of warfarin absorbed. [18] The calculated absorption rate was 92.8 % for a 10 mg dose and 96.1 % for a 2.5 mg dose. [18] In the same patient they used high performance liquid chromatography (HPLC) to measure serum warfarin concentrations to enable the calculation of the peak serum concentration and half-life. The estimated time to peak serum concentration in the patient following oral administration of warfarin was 0.5 h which is slightly faster compared to the 60–90 min reported in the early published studies of warfarin. [18] The half-life was 12.1 h which is shorter than the mean half-life but within the range reported for most studies using HPLC assays. [18].

Kearns et al. also demonstrated that a patient with 30 cm of jejunum had bioavailability consistent with that of normal individuals. [21] In this case the patient had been established on warfarin therapy for several months and was given 25 mg of subcutaneous vitamin K to normalise their INR to facilitate placement of a Broviac catheter. Warfarin was restarted, however, the patient’s INR failed to prolong after several weeks of therapy. The patient’s stool was collected over a 24-hour period and the warfarin concentration was measured using HPLC to establish if decreased absorption accounted for warfarin failure. The stools contained less than 0.05 ug/mL (limit of sensitivity) of warfarin which essentially constituted full absorption of the administered dose. [21] In this instance the authors felt that warfarin resistance was due to exogenous administration of vitamin K.

Where the residual length of small bowel was documented, patients had a small bowel length ranging from 12 cm to 100 cm. All patients apart from two had an intact duodenum. The one patient that did not have a duodenum and yet still manged to achieve a therapeutic INR had undergone complete small bowel resection and formation of a venting gastrostomy. [24] To facilitate adequate absorption the patient was advised not to vent the gastrostomy for 2–4 h after taking warfarin. [24] The successful anticoagulation of patients with even only a very small amount of residual small bowel is consistent with the previously described absorption mechanism of warfarin from the proximal small bowel.

Twenty five of the thirty one cases were on long term parenteral nutrition and an additional one on intravenous fluids with electrolytes. [6], [17], [19], [20], [22], [24], [25], [26], [27], [28], [29] Three cases in the literature highlighted the interaction between intravenous or subcutaneous vitamin K and warfarin. [19], [21], [22] Lutomski et al. reported two cases in which the usual weekly intravenous phytomenadione was not given alongside their long-term parenteral nutrition. [19] In both of these cases their warfarin requirements decreased significantly after several months of therapy which they hypothesised was due to depletion of endogenous Vitamin K stores. [19] There was another case report in which the patient was resistant to warfarin due to concomitant administration of intravenous vitamin K alongside their parenteral nutrition. [22] Following cessation of intravenous vitamin K the patient was rechallenged with warfarin and achieved a therapeutic INR. [22].

A single case of gastrointestinal haemorrhage was reported as a direct complication of warfarin therapy [17] and a second study reported 2 gastrointestinal bleeds in patients with warfarin. [27] There were no recorded fatalities attributable to warfarin. As previously described Lopez et al. reported a case of deep vein thrombosis in a patient with short bowel syndrome who was on warfarin who had a documented therapeutic INR at least 75 % of the time. [23] Fumagalli et al. [27] detailed incidence rates (defined as episodes per 100 patient years) of arterial thrombosis, venous thrombosis, bleeding, and mortality related to anticoagulation in patients with short bowel syndrome on warfarin as 0, 2, 3.4 and 0 respectively.

**Table 2:** **Summary of characteristics of the studied oral anticoagulant and antiplatelet agents.** Further details and references are contained within the main text. Of note, although INR correlates with clinical efficacy, the concentration of direct oral anticoagulants and tests of platelet function are indirect markers of drug activity but do not have known direct correlation with efficacy.

	Mechanism of action	Details of absorption	Likely site of absorption	Shortest reported small bowel length for absorption	Evidence of efficacy in short bowel syndrome
Warfarin	Vitamin K antagonist	Water soluble, passive absorption, concentration dependent	Duodenum and/or proximal jejunum	12 cm	Yes: Therapeutic INR achieved and maintained
Apixaban	Direct factor Xa inhibitor	pH independent, linear pharmacokinetics	Duodenum and/or proximal jejunum	Duodenum + 30 cm	Yes (limited): Indirect evidence with comparable plasma concentrations to population control
Rivaroxaban	Direct factor Xa inhibitor	pH independent, dose-dependent factor Xa inhibition, high bioavailability	Stomach and duodenum and/or proximal jejunum	35 cm	Yes: Indirect evidence with comparable plasma concentrations to population control
Dabigatran	Direct thrombin inhibitor	Some impact of gastric pH on Cmax but not of clinical relevance	Duodenum and/or proximal jejunum	35 cm	Indirect evidence of plasma concentrations suggest unlikely to be effective
Aspirin	COX1 inhibition with reduction of thromboxane A2	Rapid absorption	Duodenum and/or proximal jejunum	Duodenum + 30 cm	Limited indirect evidence of acceptable platelet inhibition
Clopidogrel	P2Y_12_ inhibitor	Rapid absorption	Duodenum and/or proximal jejunum	Duodenum + 30 cm	Limited indirect evidence of acceptable platelet inhibition
Ticagrelor	P2Y_12_ inhibitor	Rapid absorption, linear pharmacokinetics	Duodenum and/or proximal jejunum	Duodenum + 30 cm	Limited indirect evidence of acceptable platelet inhibition
Prasugrel	P2Y_12_ inhibitor	Rapid absorption, can be administered without regard to food but loading in the fasted state may provide more rapid onset of action, caution in weight < 60 kg	Duodenum and/or proximal jejunum	Duodenum + 30 cm	Limited indirect evidence of acceptable platelet inhibition

There was only one case of true warfarin resistance reported by Brophy et al. in which the patient did not achieve a therapeutic INR despite dose escalation. [29] The patient had duodenectomy and partial gastrectomy following multiple gunshot wounds to the abdomen. [29] The authors hypothesised that the warfarin resistance was due to a lack of a duodenum and loss of gastric tissue leading to reduced absorption. [29] A second case, not included in the systematic review due to failure to meet eligibility criteria, also described warfarin resistance in a patient with short bowel syndrome on parenteral nutrition due to supratherapeutic Vitamin K administration. [30].

#### Conclusion

There is significant patient heterogeneity within the case reports, however, the literature suggests that patients with SBS can be successfully anticoagulated with warfarin. Patients with as little 12 cm of small bowel managed to achieve a therapeutic INR. Given its favourable absorption profile, warfarin remains a relatively safe option for anticoagulation in patients with SBS despite its limitations.

### Heparin: unfractionated and LMWH

Our literature search only identified one case report specifically addressing the efficacy of intravenous unfractionated heparin therapy in a patient with SBS. [31] However, it should be noted that many of the patients in the case reports published on warfarin were on intravenous unfractionated heparin prior to initiation of warfarin therapy. The case report describes a patient with antiphospholipid syndrome who developed an arterial thrombus of the superior mesenteric artery. The patient underwent an extended enterocolectomy from the upper jejunum to the transverse colon. Post-operatively they were commenced on intravenous heparin. The APTT could not be used to monitor heparin because of a baseline prolongation in APTT due to the lupus anticoagulant, and so heparin anti-Xa levels were used. A heparin plasma concentration of 0.4 U/mL was achieved which is in keeping with reported therapeutic range of 0.2–0.4 U/mL. [31] This is not surprising as the parenteral route of administration should not be affected by short gut length.

Whilst there are publications detailing the use of LMWH in patients with intestinal failure these do not specifically address the question posed in our search strategy about efficacy in SBS specifically and so were either not identified by our search strategy or were excluded.

### Apixaban

#### Class of drug and mode of absorption

Apixaban is an oral direct factor Xa inhibitor that inhibits both free and clot bound factor Xa. [32] In healthy subjects, the absorption of apixaban appears to be primarily in the proximal gastrointestinal tract with absorption continuing as it progresses through the gastrointestinal tract. [33] Byon et al. investigated the regional gastrointestinal absorption in healthy subjects. [33] Through the use of Enterion™ capsules, the effect of a 2.5 mg dose of apixaban administered orally was assessed in the distal small intestine and the ascending colon. Cmax and area under the plasma concentration-time curve from zero to the time of the last quantifiable concentration were then measured. They found that when compared to oral administration the bioavailability of 2.5 mg of apixaban was approximately 60 % and 84 % lower when released in the distal small bowel and ascending colon respectively. [33] The oral bioavailability of apixaban is approximately 50 % with food having no clinically significant impact on absorption. [34] Absorption also occurs in a pH independent fashion. [35] This means that medications such as proton pump inhibitors which patients with SBS are frequently prescribed should not interfere with absorption.

Apixaban exhibits linear pharmacokinetics between doses of 2.5 mg and 25 mg. [34], [36] In normal healthy subjects peak plasma concentrations occur 3–4 h after oral administration with a half-life of 12 h. [36] Apixaban is metabolised by CYP3A4/5 and is a substrate for p-glycoprotein. [37] Apixaban therefore has the potential to interact with potent inhibitors or inducers of these pathways. However, there have been few clinically significant CYP/p-glycoprotein interactions reported in the literature and most of the clinically relevant interactions which result in increased bleeding risk are from the additive effects of another medication, such as an antiplatelet or non-steroidal anti-inflammatory agent. [37].

#### Discussion of apixaban studies

There was one case report in the literature of anticoagulation in a 45-year-old patient with SBS following extensive gut resection after a mesenteric thrombosis (it was not documented if thrombus was venous or arterial) using apixaban. [38] The patient was taking daily fondaparinux injections after developing heparin induced thrombocytopenia. Due to the financial cost of fondaparinux the authors explored the option of commencing the patient on a DOAC. They hypothesised that the absorption profile and linear pharmacokinetics of apixaban would mean that they could evaluate the patient’s personal pharmacokinetics and then establish a personalised dosing regimen for the patient. Efficacy of the dosing regimen could subsequently be confirmed by analysing plasma level. They developed a three-step protocol which first involved establishing apixaban absorption in the patient whilst continuing therapeutic anticoagulation with fondaparinux. The peak plasma concentration measured in the patient was compared to median steady state peak plasma concentration in healthy subjects taking 2.5 mg twice daily for non-valvular atrial fibrillation. [11] A threshold of greater than or equal to 20 % of the median steady state peak plasma concentration was set by the investigators as representing sufficient absorption to progress onto the next step, which involved calculation of personalised dosing. The patient’s peak plasma concentration was 30 % of the population median so the investigators proceeded to calculate a personalised twice daily apixaban dose. They settled on a dose of 15 mg twice daily. The patient was administered 15 mg of apixaban twice daily for three doses. Trough and peak plasma concentrations were obtained before and after the third dose. The patient achieved peak plasma concentrations (316 ng/mL) approaching the 95th percentile seen in normal patients. In the third step the patient was continued on apixaban long term with peak plasma concentrations measured after one month and five months of therapy. The patient achieved peak plasma concentrations within the targeted range (100–300 ng/mL) on both occasions and did not have any episodes of recurrent thrombosis or any reported bleeding events over the 5-month period of follow-up.

This case study demonstrates that apixaban can be used in a patient with SBS with personalised dosing based on measuring peak plasma concentrations and aiming to keep them within an expected ‘peak’ range. Over a short duration of follow-up this patient did not experience any known problems from this individualised apixaban dosing regimen, including bleeding or recurrent thrombosis. It is, however, unclear how much residual small bowel the patient had and therefore difficult to know if patients with a total enterectomy or minimal residual small bowel would achieve sufficient absorption.

#### Conclusion

The approach outlined by the authors could be considered for similar patients following careful counselling with regards to lack of known efficacy, in those who are intolerant or poorly controlled on warfarin. Further research is required and should include apixaban levels and clinical outcomes. A clinical trial of pharmacokinetics of apixaban in patients with short bowel syndrome is in progress (Clinicaltrials.gov identifier: NCT04344717).

### Rivaroxaban

#### Class of drug and mode of absorption

Rivaroxaban is an oral direct Factor Xa inhibitor that inhibits both free and clot bound Factor Xa which results in inhibition of thrombin generation. [13] The absorption of rivaroxaban appears to be primarily in the stomach and proximal small intestine. [14] It has high bioavailability with greater than 80 % of a 10 mg dose being readily absorbed after oral administration, irrespective of fasting or fed conditions. [13] At higher doses absorption is improved by administration with food. [13] Peak plasma concentrations are reached within 2–4 h of dosing. [35] Factor Xa activity is inhibited in a dose dependent manner, peaking approximately 3 h after oral administration and continuing until the end of the dosing interval. [39].

Rivaroxaban is metabolised by the CYP3A4/5 and CYP2J2 enzymes, in theory making it susceptible to cytochrome P450 drug interactions. [40] In reality there are very few clinically significant drug interactions which have been identified. Of particular relevance to patients with SBS, the pharmacokinetic parameters of rivaroxaban are not affected by changes in gastric pH induced by ranitidine or omeprazole. [39], [41].

#### Discussion of rivaroxaban studies

Douros et al. described a case of successful anticoagulation using rivaroxaban in a patient with 100 cm of intact small bowel following a right cardioembolic middle cerebral artery stroke. Prior to the stroke the patient was taking dabigatran. Because the measured levels of dabigatran were lower than expected (see further discussion in dabigatran section) and the patient was at high risk of recurrent thrombosis, rivaroxaban 20 mg daily was trialled. Following taking rivaroxaban for twelve weeks the peak plasma concentration (Cmax, 223 μg/L) was measured 2.5 h after ingestion and found to in normal range(180–405 μg/L) documented for patients taking 20 mg for AF. [42].

These findings were consistent with those of Cheung et al. who undertook an interventional crossover study comparing the pharmacokinetics of dabigatran and rivaroxaban in six patients with SBS. [2] Patients took either dabigatran 150 mg twice daily or rivaroxaban 20 mg daily for five days followed by a washout period of at least four days. They were then switched to the other direct oral anticoagulant. Bloods samples were obtained in fasting state on day 0 at time 0 and three hours. On day 4 blood samples were obtained at time 0 (pre-dose) 1, 2, 3, 4, 5, 6, 8, 10, 12 (dabigatran only) and 24 (rivaroxaban only) hours following the last dose. The plasma concentrations at the different time intervals were then used to calculate several different pharmacokinetic parameters including Ctrough, Cmax, AUC_0–1_ and clearance of the drug after oral administration. These values were then compared to reference values obtained from studies of patients receiving rivaroxaban 20 mg once daily to obtain mean observed to reference ratios. The mean observed to reference ratios for rivaroxaban were overall much higher than dabigatran. [2] When compared to patients taking rivaroxaban for atrial fibrillation the mean Cmax_ss_ ratio was 0.76. [2] When compared to patients taking rivaroxaban for deep vein thrombosis the mean Cmax_ss_ ratio was 0.70. [2].

In addition to the two papers previously described there were another two articles examining rivaroxaban use in patients with SBS. Christensen et al. presented a case series of three patients with SBS who were successfully anticoagulated with rivaroxaban. [43] Indications for anticoagulation were mesenteric vein thrombosis, catheter-related venous thrombosis and DVT. The length of residual small bowel in the patients ranged from 30–100 cm. All of the subjects were established on long term parenteral nutrition. Patients received rivaroxaban 20 mg daily and plasma concentrations were measured within 2–4 h of oral administration to obtain a Cmax level. The authors report that the Cmax values for all three patients were within the 90 % central interval for concentrations found for patients receiving 20 mg rivaroxaban once a day in a phase II clinical trial of rivaroxaban. [43], [44] One patient had to stop rivaroxaban therapy due to intolerance with severe nausea. The other two patients were continued on rivaroxaban and had no thrombotic complications at six and twelve months respectively. [43].

Bavalia et al. undertook a prospective observational cohort study to examine the safety and efficacy of rivaroxaban in patients with SBS. [45] Patients were commenced on rivaroxaban 20 mg once daily and underwent a pharmacokinetic analysis within 2 weeks. Those that demonstrated sufficient peak plasma levels (Cmax) in comparison to the reference range published in phase II trials (189–419 ng/mL)^45^ were allowed to continue and were followed up clinically. The pharmacokinetic analysis was repeated at three and six months. Five patients with SBS on long term parenteral nutrition were enrolled in the study. Where known the residual length of small bowel ranged from 45 to 120 cm. The indication for anticoagulation was either atrial fibrillation or venous thromboembolism. Three of the five patients had Cmax levels within the expected reference range. The other two patients had levels below this range and therefore did not continue in the study. The three patients who continued on rivaroxaban were followed up for a median of five months. None of the patients had a major bleeding event. However, one of the patients who was taking anticoagulation for a previous VTE and whom had a Cmax in the reference range at visit 1 (approximately 220 ng/mL) developed a pulmonary embolus (PE). [45] This patient was defined as having intestinal failure due to refractory obstipation and it is unknown if they had previous bowel resections. It is difficult to know how to interpret this event because repeat Cmax levels are the time of diagnosis of the PE were not provided and the authors do not comment on patient compliance.

As a presumed extension of the dataset published by Bavalia et al., van Haaps et al. [46] report preliminary findings of the TINCRBEL study (EudraCT number 2018–001845-15). Seventeen patients with intestinal failure requiring parenteral nutrition (10 with short bowel syndrome) and commencing rivaroxaban 20 mg once daily underwent a 6-point pharmacokinetic profile within 2 weeks with results compared with reference ranges from patients without intestinal failure. Those with sufficient absorption continued on rivaroxaban as could those with subtherapeutic results but only as part of shared decision making with the patient. 14 patients (82 %) continued therapy with no reported thrombosis or major bleeding (cumulative 22.5 year follow up).

#### Conclusion

Although very limited, the current evidence to date could suggest that rivaroxaban may be an effective anticoagulant in patients with SBS with as little as 30 cm of residual small bowel (negligible absorption was seen with only 15 cm residual small bowel). Nearly all the cases achieved satisfactory peak plasma concentrations (Cmax) in comparison to population data obtained in the original phase I, II and III studies. [13] However one patient did develop a PE despite having a Cmax level in the expected range when it was first tested two weeks after commencing rivaroxaban. Of course, patient compliance and the Cmax level at the time of diagnosis of the PE is unknown. A further one patient had to stop therapy due to severe nausea and no patients were reported to have had a significant bleeding event. Further research is certainly required to better establish safety and efficacy of rivaroxaban in patients with SBS. This needs to be conducted with careful patient consent/counselling and close monitoring. The outcome of the TINCRBEL study may be helpful in better understanding pharmacokinetics and clinical outcomes for rivaroxaban in intestinal failure. This is expected to report in 2025 (personal communication).

### Dabigatran

#### Class of drug and mode of absorption

Dabigatran is a reversible direct thrombin inhibitor. It is administered as a prodrug called dabigatran etexilate which is cleaved by serum and hepatic esterase into its active form. [35] The proprietary formulation of dabigatran etexilate capsules contain tartaric acid spherules which help reduce the variability of its absorption which is dependent on an acidic environment. [35], [47] As a result, the absorption of dabigatran is independent of gastrointestinal tract acidity. Co-administration of proton pump inhibitors has been found to reduce the bioavailability of dabigatran with Cmax values being 28 % less when compared to subjects not taking proton pump inhibitors. [48] However, this reduction in Cmax was not thought to be clinically relevant and dose adjustment was deemed unnecessary. [48].

Dabigatran capsules are designed for release in the stomach and absorption is thought to occur primarily in the proximal small intestine. [35] Administration with food does not impact on absorption of the drug. [12] Peak plasma concentrations are reached approximately 2 h after administration in healthy volunteers. [48] Dabigatran is not metabolised by CYP4 enzymes and therefore has a low potential for drug interactions. [40] The pro-drug dabigatran etexilate is a substrate for p-glycoprotein, as such it could theoretically interact with potent inhibitors or inducers of this transporter. [40] However, this is unlikely to be relevant in patients with SBS as p-glycoprotein is most abundantly expressed in the distal small bowel and most patients with SBS have undergone extensive surgical resection of this portion of bowel.

#### Discussion of dabigatran studies

There are two case reports in the literature in which dabigatran has been administered to patients with SBS. [2], [42] As previously described Douros et al. reported a case of a right cardioembolic middle cerebral artery stroke in a patient with 100 cm intact small bowel who was taking dabigatran 110 mg twice daily for three months prior to presentation. [42] Peak and trough concentrations of dabigatran were measured using a thrombin inhibitor assay in order to help determine whether the patient was achieving dabigatran levels within the expected range. Both the peak and trough plasma concentrations of dabigatran were substantially lower compared to the values measured in patients with the same creatinine clearance in the RE-LY trial. [42] The authors hypothesised that the failure of dabigatran in this instance was a result of poor absorption from the gut. Although detailed knowledge about the exact absorption site of dabigatran is currently lacking, dabigatran etexilate is a P-gp substrate and this is predominantly expressed in the jejunum and ileum which may account for failure of dabigatran in this patient.

The findings of Douros et al. are consistent with Cheung et al., who undertook an interventional crossover study comparing the pharmacokinetics of dabigatran and rivaroxaban in six patients with SBS. [2] The methodology is outlined in the previous section. The Ctrough and Cmax values found in patients taking dabigatran were lower than those observed in the phase III RE-LY trial. [2], [49] The mean Cmax_ss_ ratio was 0.57 and the mean Ctrough_ss_ ratio was 0.35. [2].

#### Conclusion

Studies demonstrate that dabigatran is absorbed to some extent in patients with SBS however there was significant inter-individual variability in the plasma concentrations observed. This almost certainly reflects significant heterogeneity amongst the study population. The plasma concentrations of dabigatran observed at steady state were lower than the reference values obtained from phase II and III trials, therefore, dabigatran may not represent the best choice of DOAC in the SBS population.

### Antiplatelets

#### Class of drug and mode of absorption

Aspirin irreversibly binds to cyclo-oxygenase 1 which results in decreased production of thromboxane A2 which is responsible for amplification of platelet aggregation. [50] It is rapidly absorbed from the proximal gastrointestinal tract. [50] Unlike aspirin the newer antiplatelet agents including clopidogrel, ticagrelor and prasugrel are PY_12_ receptor inhibitors. The PY_12_ receptor plays a key role in adenosine diphosphate stimulation of the glycoprotein IIa/IIIb receptor which results in platelet degranulation and thromboxane production. [51] The different PY_12_ inhibitors have significant pharmacokinetic differences. Both prasugrel and clopidogrel are prodrugs that need to be converted to an active drug by several different metabolic pathways. [52] Clopidogrel is converted to its active metabolite via a two-step hepatic cytochrome P450 pathway which is highly polymorphic. [52], [53] Similarly prasugrel requires enzymatic activation via cytochrome dependent pathway. [53] Ticagrelor does not require enzymatic activation, however, it is a substrate/inhibitor of CYP3A4 and is therefore susceptible to drug interactions. [53] Our systematic review identified three papers looking at antiplatelet use in SBS.

#### Discussion of aspirin monotherapy studies

Faye et al. evaluated the efficacy of low dose aspirin in ten patients with SBS secondary to mesenteric ischaemia. [54] The mean small bowel length of the study participants was 70 + /- 46 cm. Low dose aspirin(75–160 mg) was administered for at least seven days and then blood samples were collected 24 h (T0) after the last aspirin dose and one hour post aspirin administration (T1, equates to Tmax). Nine of the patients took aspirin orally and one patient received it intravenously as a control. Platelet aggregation was measured by light transmission aggregometry within 2 h of blood sampling to determine the inhibition of platelet prostaglandin pathways by aspirin. Platelet aggregation was induced exogenously, and the maximum aggregation intensity (MAI) was determined. Maximal aggregation intensity is the maximal percentage change in light transmittance from baseline using platelet poor plasma as a reference. The investigators considered patients to be aspirin resistant when the MAI was > 20 %.

One hour post aspirin administration (T1, Tmax) the mean MAI was 7.6 % + /- 4.5 %, meaning that no patients were aspirin resistant. This suggests that all patients achieved adequate absorption of aspirin. Twenty-four hours (T0) post aspirin administration 3 out of 10 patients were considered to be aspirin resistant because they had MAI greater than 20 %. The mean MAI was 22.7 % + /- 22 % (range 4–69). [54].

The investigators undertook some additional analysis including assessing platelet reactivity using a platelet function analyser. In this test blood is forced through a collagen coated membrane preactivated with epinephrine which generates high shear stress and activates primary haemostasis. The time it takes for the aperture in the membrane to become occluded is defined as the closure time (CT). CT is prolonged in patients taking aspirin and the investigators used a cut-off value of CT > 160 s to define responders to aspirin. One hour post aspirin administration (T1) all patients had normal impaired platelet reactivity (CT >160 s). [54] 24 h post aspirin administration (T0) the mean CT was 210 + /- 67 s [54] Three out of ten patients had a CT less then 160 and therefore were deemed resistant to aspirin. [54] One of the patients (patient 2) at T0 had both a CT (145 s) and MAI (59 %) in keeping with aspirin resistance. [54] In four patients at T0 (patients 3, 7, 9, 10) the results of the light aggregometry and platelet reactivity were not concordant. [54] However, platelet function testing to quantify response to aspirin is controversial. [55] There have been several studies which have reported poor correlation between the two assays. [55] Therefore, this result must be interpreted with caution. Although light transmission aggregometry is considered the gold standard for evaluating response to aspirin in the research setting, in reality it is used rarely in clinical practice. [55].

This study demonstrates that sufficient aspirin absorption was achieved one hour post oral administration in all patients when measured by light transmission aggregometry. [54] The residual length of small bowel or dose of aspirin did not appear to influence the results. The cause for aspirin resistance observed in three patients at 24 h is unclear, however, it must be independent of residual small bowel length. The authors hypothesise that this could be explained by a number of factors including increased platelet turnover, drug interactions, upregulated sources of thromboxane biosynthesis and genetic polymorphisms of COX1. [54] It has previously been reported that once daily aspirin does not provide stable 24 h antiplatelet protection when measured by light transmission aggregometry in a significant proportion of patients (28 %) with coronary artery disease. [56].

#### Discussion of dual antiplatelet and P2Y12 antiplatelet studies

Hadley et al. presented a case of a patient with SBS who had single vessel coronary artery disease demonstrated on coronary angiography and underwent placement of a bare metal stent. [57] Following stent insertion, the patient was commenced on dual antiplatelet therapy with aspirin and clopidogrel. Antiplatelet response for both aspirin and clopidogrel was assessed using light transmission aggregometry and whole blood impendence aggregometry. Both assays confirmed aspirin responsiveness but response to clopidogrel was only partial when assessed by light transmission aggregometry and absent when assessed by whole blood impedance aggregometry. [57] The patient was then switched from clopidogrel to ticagrelor, and the light transmission aggregometry repeated. This demonstrated acceptable platelet inhibition on ticagrelor. [57] It should be noted that the authors do not define thresholds for aspirin responsiveness for both assays and the timing of the blood samples post oral administration is not documented. The patient’s residual length of small bowel is also not included. Variable response to clopidogrel is well recognised and can be genetic due to CYP P450 2C19 polymorphisms or acquired in patients with diabetes or renal failure. [57] Ticagrelor may be an effective alternative in patients who are hyporesponsive to clopidogrel.

Droppa et al. developed an individualised dual antiplatelet regimen in a patient with SBS by assessing antiplatelet response using multiple electrode aggregometry. [52] The patient had SBS secondary to occlusion of the superior mesenteric artery and was left with an intact duodenum and 30 cm of jejunum following surgical resection. The patient had a subsequent anterior ST-segment myocardial infarction and underwent placement of three bare metal coronary artery stents necessitating administration of dual antiplatelet therapy to prevent in stent thrombosis. [52] The patient was initially commenced on a combination of aspirin and clopidogrel. Due to concern regarding insufficient absorption in the context of SBS the antiplatelet effect was assessed by multiple electrode aggregometry (MEA). In multiple electrode aggregometry platelet aggregation in whole blood is stimulated between two electrodes and the electrical impedance measured. [58] A MEA value of < 46 aggregation units was considered an adequate response. [52] The patient was aspirin responsive with a MEA of 13 aggregation units but was nonresponsive to clopidogrel with 73 aggregation units. The dose of clopidogrel was increased to 75 mg twice daily and repeat MEA was performed after five days, once steady state should have been reached. However, despite the dose increase the patient remained nonresponsive to clopidogrel with a MEA of 70 aggregation units. The patient was subsequently switched from clopidogrel to prasugrel with a loading dose of 60 mg followed by 10 mg daily. After five days MEA was performed revealing that the patient had an insufficient response to prasugrel with a MEA of 64 aggregation units. Consequently, the patient was changed to ticagrelor 90 mg twice daily with a loading dose of 180 mg. After five days of ticagrelor therapy MEA demonstrated that the patient had an adequate antiplatelet response with a MEA of 35 aggregation units.

Allen et al. [59] describe the use of ticagrelor following coronary artery stenting in a patient with previous roux-en-Y bypass surgery for duodenal carcinoid disease. Although the length of small bowel in continuity is not detailed and therefore short bowel syndrome cannot necessarily be inferred, platelet aggregation studies did demonstrate adequate platelet inhibition in this patient (personal communication).

#### Conclusion

To date the literature regarding antiplatelet use in SBS relies on platelet function testing to establish efficacy, but this is not widely used in clinical practice because results between assays are variable and they have not been shown to correlate with clinical outcome. [60] None of the studies detailed here looked at clinical outcomes such as in stent thrombosis or recurrent ischaemic events. Moreover, it is notable in the literature that so many patients with mesenteric ischaemic events resulting in short bowel syndrome are managed with anticoagulants rather than antiplatelet agents and this may represent the dearth of available evidence. However, it should be recognised that the requirement for anticoagulation versus antiplatelet agents will differ based on the underlying aetiology of mesenteric ischaemia, Longer term studies that focus on clinical outcomes are needed to understand the efficacy of antiplatelets and the management of cardiovascular risk in this population.

## Conclusion

Drug therapy in patients with SBS represents a therapeutic challenge due to limited surface area being available for absorption. There is a paucity of high-quality literature regarding efficacy and clinical outcomes of SBS patients being treated with anticoagulants and antiplatelet agents. The evidence base predominantly consists of case reports and a sparse number of cohort studies with limited study populations. Therefore, it is difficult to generalise the findings to the larger SBS population. This is further complicated by substantial heterogeneity amongst the SBS population. However, based on the evidence presented in this systematic review it is possible to make some informed recommendations.

Despite its limitations, warfarin remains a valid oral anticoagulant in patients with SBS, especially if initiated several months post-surgical resection when the small bowel has undergone a period of adaptation. Its proximal absorption profile means that patients with as little as 12 cm of small bowel have been successfully anticoagulated. [20] In one case a patient with a complete small bowel resection and venting gastrostomy still managed to achieve a therapeutic INR with clamping of the gastrostomy for 2–4 h post-dose. [24] Although often cited as a downfall, monitoring of the patients INR means that clinicians can be reassured that a patient is achieving a therapeutic level of anticoagulation.

Regarding the newer DOACs, rivaroxaban has the most evidence to support its use in patients with SBS. It’s high bioavailability and proximal absorption profile overcome the physiologic barrier posed by SBS. Nearly all the cases presented in this review who were administered rivaroxaban achieved satisfactory peak plasma concentration in comparison to population data. [2], [42], [43], [45] Although measuring plasma levels of DOAC’s is a helpful method of response assessment it should be noted that there is no data linking plasma levels with clinical efficacy. Long term studies which focus on clinical outcomes and safety are needed.

There is limited published evidence assessing antiplatelet efficacy based on laboratory testing in patients with SBS. All the cases presented in this review demonstrated an adequate antiplatelet response to aspirin within one hour of administration when measured by light transmission aggregometry or multiple electrode aggregometry. [52], [54], [57] The rate of non-responders 24 h post aspirin dosing was comparable to that demonstrated in normal patients with coronary artery disease. [56] If a patient with SBS requires a second antiplatelet agent, based on the available evidence, ticagrelor is likely the pragmatic choice as it does not require metabolic activation via the CYP450 pathway and therefore is not susceptible to genetic polymorphisms. However it should be noted that platelet function testing is rarely used in clinical practice due to discrepancy between assay results and lack of established correlation with clinical efficacy. [60].

Anticoagulation and antiplatelet therapy in SBS remain a challenge due to concerns about absorption and the limited evidence base for efficacy of the DOACs in particular. Counselling patients with regards to this to support an informed choice is crucial. The advent of gut hormone therapy (e.g. teduglutide) to enhance small bowel absorption may result in altered drug absorption profiles and this cohort will be important to study. Future research also needs to focus on longer term clinical outcomes to improve the management of cardiovascular risk factors in the SBS population.

## Funding statement

No funding was required to undertake this work.

## Ethical statement

This work did not involve the use of human subjects and synthesises already published data. This work did not involve animal experiments.

## CRediT authorship contribution statement

**Carolyn Mercer:** Writing – original draft, Methodology, Formal analysis, Data curation. **Anna Crawford:** Writing – review & editing, Methodology, Data curation. **Susan Shapiro:** Writing – review & editing. **Philip J Allan:** Writing – review & editing, Data curation. **Tim Ambrose:** Writing – review & editing, Supervision, Methodology, Formal analysis, Data curation, Conceptualization.

## Declaration of Competing Interest

CM: No conflicts of interest to declare.

AC: No conflicts of interest to declare.

SS: SS has received conference support and educational speaker fees from Bayer, and educational speaker fees and advisory board fees from Pfizer.

PA: No conflicts of interest to declare.

TA: No conflicts of interest to declare.
