# Supplementary material for: Anticoagulation and antiplatelet therapy in short bowel syndrome: A systematic review
Source: Intest Fail. 2024 Jul 15;1:100007. doi: 10.1016/j.intf.2024.100007 (PMC12851339; doi:10.1016/j.intf.2024.100007)
Supplement: Supplementary file 1 — Supplementary material [file mmc1.docx]

**Supplementary Methods**

**Search Strategy for MEDLINE**

Medline (Ovid MEDLINE® Epub Ahead of Print, In-Process & Other Non-Indexed Citations, Ovid MEDLINE® Daily and Ovid MEDLINE®) 1946 to present

| 1 | Short Bowel Syndrome/ |
| --- | --- |
| 2 | Intestine, Small/ and Malabsorption Syndrome/ |
| 3 | exp enterostomy/ or gastrostomy/ or pancreaticoduodenectomy/ or pancreaticojejunostomy/ |
| 4 | ((short bowel or short gut) adj3 syndrome?).ti,ab,kw. |
| 5 | ((small bowel or small gut or small intestine) adj3 syndrome?).ti,ab,kw. |
| 6 | (gastrostom* or duodenostom* or jejunostom* or jejeunostom* or ileostom* or stoma?).ti,ab,kw. |
| 7 | 1 or 2 or 3 or 4 or 5 or 6 |
| 8 | exp Anticoagulants/ |
| 9 | ((anticoag* or anti-coag* or antithrombo* or anti-thrombo*) adj2 (agent? or drug? or therap*)).ti,ab,kw. |
| [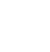](http://ezproxy-prd.bodleian.ox.ac.uk:2843/sp-4.06.0a/ovidweb.cgi?&S=NMDLFPNNIPEBJCADIPBKDHPEJKGGAA00&R=11&Search+Annotations+Options=SA)10 | (aspirin or warfarin or heparin* or coumarin* or vitamin k antagonist* or direct thrombin inhibitor* or clopidogrel or ticagrelor or fondaparinux or acenocoumarol or phenindione or direct factor xa inhibitor* or noac or doac or bivalirudin or argatroban or desirudin or dabigatran or rivaroxaban or edoxaban or betrixaban or apixaban).ti,ab,kw. |
| 11 | (anticoag* or anti-coag* or antithrombo* or anti-thrombo*).ti. |
| 12 | 8 or 9 or 10 or 11 |
| 13 | 7 and 12 |
| 14 | exp animals/ not humans.sh. |

**Search Strategy for EMBASE**

Embase 1974 to present

| [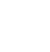](http://ezproxy-prd.bodleian.ox.ac.uk:2843/sp-4.06.0a/ovidweb.cgi?&S=GDKMFPLNHGEBJCOOIPBKNEPEDDMFAA00&R=9&Search+Annotations+Options=SA)1 | Short Bowel Syndrome/ |
| --- | --- |
| 2 | exp enterostomy/ or gastrostomy/ |
| 3 | ((short bowel or short gut) adj3 syndrome?).ti,ab,kw. |
| 4 | ((small bowel or small gut or small intestine) adj3 syndrome?).ti,ab,kw. |
| 5 | (gastrostom* or duodenostom* or jejunostom* or jejeunostom* or ileostom* or stoma?).ti,ab,kw. |
| 6 | 1 or 2 or 3 or 4 or 5 |
| 7 | exp *anticoagulant agent/ |
| 8 | ((anticoag* or anti-coag* or antithrombo* or anti-thrombo*) adj2 (agent? or drug? or therap*)).ti,ab,kw. |
| 9 | (aspirin or warfarin or heparin* or coumarin* or vitamin k antagonist* or direct thrombin inhibitor* or clopidogrel or ticagrelor or fondaparinux or acenocoumarol or phenindione or direct factor xa inhibitor* or noac or doac or bivalirudin or argatroban or desirudin or dabigatran or rivaroxaban or edoxaban or betrixaban or apixaban).ti,ab,kw. |
| 10 | (anticoag* or anti-coag* or antithrombo* or anti-thrombo*).ti. |
| 11 | 7 or 8 or 9 or 10 |
| 12 | 6 and 11 |
| 13 | (exp animals/ or nonhuman/) not human/ |
| 14 | 12 not 13 |

Search Strategy for CENTRAL

| #1 | MeSH descriptor: [Short Bowel Syndrome] explode all trees |
| --- | --- |
| #2 | MeSH descriptor: [Malabsorption Syndromes] this term only |
| #3 | MeSH descriptor: [Enterostomy] explode all trees |
| #4 | MeSH descriptor: [Gastrostomy] this term only |
| #5 | ((("short bowel "or "short gut") NEAR/3 syndrome*)):ti,ab,kw OR ((("small bowel" or "small gut" or "small intestine") NEAR/3 syndrome*)):ti,ab,kw OR (gastrostom* or duodenostom* or jejunostom* or jejeunostom* or ileostom* or stoma or stomas):ti,ab,kw |
| #6 | #1 or #2 or #3 or #4 or #5 |
| #7 | MeSH descriptor: [Anticoagulants] explode all trees |
| #8 | (((anticoag* or anti-coag* or antithrombo* or anti-thrombo*) NEAR/2 (agent* or drug* or therap*))):ti,ab,kw OR ((aspirin or warfarin or heparin* or coumarin* or vitamin k antagonist* or direct thrombin inhibitor* or clopidogrel or ticagrelor or fondaparinux or acenocoumarol or phenindione or direct factor xa inhibitor* or noac or doac or bivalirudin or argatroban or desirudin or dabigatran or rivaroxaban or edoxaban or betrixaban or apixaban)):ti,ab,kw OR ((anticoag* or anti-coag* or antithrombo* or anti-thrombo*)):ti |
| #9 | #7 or #8 |
| #10 | #6 and #9 |
